# Relationship between epicardial adipose tissue and coronary function evaluated with [13 N]NH3 PET/CT.

**DOI:** 10.1007/s10554-026-03612-0

**Published:** 2026-01-27

**Authors:** Francesco Dondi, Mattia Bertoli, Pietro Bellini, Gianluca Viganò, Luca Camoni, Roberto Rinaldi, Michela Cossandi, Massimo Statuto, Francesca Tomasoni, Enrico Vizzardi, Francesco Bertagna

**Affiliations:** 1 Nuclear Medicine, ASST Spedali Civili di Brescia and Università degli Studi di Brescia, Brescia, 25123 Italy; 2 Nuclear Medicine, ASST Spedali Civili di Brescia, Brescia, 25123 Italy; 3 Clinical Engineering, ASST Spedali Civili di Brescia, Brescia, 25123 Italy; 4 Cardiology Unit, ASST Spedali Civili di Brescia and Università degli Studi di Brescia, Brescia, 25123 Italy

**Keywords:** Epicardial adipose tissue, EAT, PET/CT, [13N]NH3, Myocardial flow reserve, Perfusion imaging

## Abstract

Different reports have suggested that epicardial adipose tissue (EAT) is related to impaired coronary function, myocardial ischemia and major adverse cardiac events. The aim of this study was to evaluate whether a relationship between EAT volume (EATvol) and thickness (EATthick) with myocardial blood flow (MBF) and myocardial flow reserve (MFR) evaluated with [^13^N]ammonia ([^13^N]NH3) positron emission tomography/computed tomography (PET/CT) is present.MBF and MFR were extracted from stress and rest [^13^N]NH3 PET/CT images. EATvol was extracted from unenhanced CT images with a semiautomatic method; EATthick from the same images. Correlation between these parameters, MBF, MFR and other clinicopathological features were investigated. Independent predictors for MBF and MFR were identified.we retrospectively enrolled 164 patients. MFR was reduced in 30 subjects. EATvol and EATthick were significantly different between subjects with normal and impaired MFR in the total cohort (p 0.001 and 0.005, respectively) and also dividing the patients on the basis of CAD history (p 0.017 and 0.038 respectively for patients without CAD, p 0.010 and 0.007 respectively for subjects with CAD). In the total cohort age, diabetes and EATvol were independent predictors of MFR (p 0.036, 0.029 and 0.008, respectively). Presence of diabetes and EATvol were confirmed as independent predictors also in the group of patients without CAD history (p 0.042 and 0.038, respectively), while in subjects with CAD history no features were confirmed. Similarly, EATvol and EATthick were not independent predictors of stress MBF.a correlation with EATvol and impaired MFR has been reported, confirming therefore the idea that EAT may influence coronary function.

## Introduction

Epicardial adipose tissue (EAT) is defined as the adipose tissue located between the myocardium and the visceral pericardium. This tissue is a metabolically active organ which plays a role as a fat store-house for the heart, producing also a different variety of bioactive molecules which could influence cardiac function. The anatomic proximity between EAT and myocardium may imply the presence of a paracrine regulation deriving from the first tissue [1].

EAT has particularly characteristics such as a high density of small adipocytes, suggesting therefore specific and peculiar metabolic properties related to a greater release of free fatty acids [2]– [3]. Furthermore, it has been demonstrated that EAT correlates with the presence of cardiac sympathetic denervation, representing also a source of catecholamine production and having therefore a potential role in cardiac response to sympathetic stimulation [4]. Additionally recent evidences suggest that the volume of the EAT may be helpful for the estimation or pretest probability of obstructive coronary artery disease (CAD) over traditional risk factors and coronary calcium score [5]. Moreover, it has been proposed that the quantification of EAT may have prognostic impact in predicting adverse cardiovascular events [6]– [7].

Myocardial perfusion imaging (MPI) is currently a milestone for the diagnosis and the management of patients affected by CAD, giving also important prognostic information useful for their management [8–10]. This modality reflects the relative difference in the distribution of blood flow in the myocardium during stress and at rest, demonstrating the presence of perfusion reduction which reflects the myocardium ability to adapt to blood supply to an increased work [9, 11]. In this scenario, positron emission tomography (PET) has been demonstrated in the past as a tool that can perform MPI with great accuracy, allowing moreover the precise measure of stress and rest myocardial blood flow (sMBF and rMBF, respectively) and myocardial flow reserve (MFR) [12]. Indeed, a reduction of MFR is related to the presence of coronary artery stenosis and to coronary vascular disfunction [13]. Furthermore, PET imaging has been demonstrated in the past as useful for the assessment of different conditions that can affect the cardiovascular system, such as atherosclerotic plaque and their inflammation processes or for the diagnosis of endocarditis [14]– [15].

Some evidences in literature have suggested the presence of a correlation between an increased presence of EAT and an impaired MFR, however this association needs to be yet fully investigated [16–19]. Moreover, these evidences have been underlined with the use of 82-rubidium ([82Rb]) PET MPI. The aim of this study is therefore to assess the relationship between EAT and coronary vascular function evaluated by [13 N]ammonia ([13 N]NH3) PET/computed tomography (PET/CT).

## Materials and methods

### Patients selection

Patients included in the study were retrieved by retrospectively screening the databases of our institution, for subjects admitted to our center to perform [^13^N]NH3 PET/CT with different indications such as for example the evaluation of chest pain of suspected cardiac origin and the assessment before and after revascularization: all patients included in the study had inconclusive imaging prior to undergoing the [^13^NNH3 PET/CT. Inclusion criteria were being adult, having underwent rest and stress [^13^N]NH3 PET/CT for myocardial perfusion assessment and completeness of clinical data necessary for analyses. Exclusion criteria were the presence of missing or incomplete clinical information required for statistical analyses. The screening period started from January 2014 and ended in April 2025. Informed consent was obtained from all individual participants included in the study by contacting them. The study adhered to the declaration of Helsinki guidelines and received approval from the local ethical committee (protocol number 0039649/25).

### [13 N]NH3 PET/CT imaging

Patients were asked to not withdraw their medications before the exam. A CT scan (120 kV, 80 mA, pitch 1 mm) was first of all acquired for optimal position on a CT scout scan and to create an attenuation correction map. [^13^N]NH3 was produced according to a previously described process [20]. To to ensure proper pharmacological stress induction, patients were instructed to avoid the use of caffeine at least 24 h before the scan, then asking them at the time of the examination whether they had followed these instructions. To obtain maximal vasodilatation, an intravenous injection of 400 mcg of regadenoson over 10 s was performed. The heart rate, systemic blood pressure, and 12-lead electrocardiogram were recorded at baseline and throughout the infusion of pharmacological stressor. Forty seconds after the end of the injection of regadenoson, a bolus administration of a standard dose of 370 MBq or 185 MBq of [^13^N]NH3, according to the protocol used, was performed. PET studies were acquired in 3D and list mode for 10 min, starting the acquisition immediately before the injection of the radiotracer. The tomograph used was a Discovery PET/CT 690 (GE Healthcare, Milwaukee, Wisconsin, USA). Images were evaluated after an iterative reconstruction of tomographic slices, following the recommendations of the American Society of Nuclear Cardiology (ASNC) [21]. Transaxial PET perfusion images were reoriented into short-axis and vertical and horizontal long-axis slices. sMBF, rMBF and MFR were calculated and determined using 4-DM Corridor software package (INVIA, Ann Arbor, Michigan) using the standard two compartments model. MFR was calculated as the ratio between sMBF and rMBF and expressed in mL/min/g. An MFR ≥ 2 was considered normal. Both MBF and MFR were evaluated for the entire left ventricle and for the three main coronary territories using a 17-segment model, according to what recommended by the ASNC [21]. For further analyses, only the values of MBF and MFR of the entire left ventricle were used.

### EAT quantification

Two experienced nuclear medicine physicians (F.D. and M.B.) analyzed the unhenanced CT images of the PET/CT scans, blinded to the results of the PET results. EAT volume (EATvol) was quantified on CT images with LIFEx 25.06.1 software (LIFEx by the French Alternative Energies and Atomic Energy Commission (CEA), Gif-sur-Yvette, France) [22]. As previously described, the images processing started at the level of the pulmonary trunk and ended at the level of the inferior diaphragmatic surface of the heart [19]. A volume of interest (VOI) was manually drawn to include all the tissues inside the pericardium and, after applying a threshold based on an attenuation range between −30 and −190 HU [23], EATvol was extracted and expressed in cm3. The final ROI was checked and reviewed by the operators to correct potential errors of segmentation and, when needed, manually adjusted in each slice [24]– [25].

Using Vue PACS software (Philips N.V., Amsterdam, The Netherlands), EAT thickness (EATthick) was measured along the right ventricle inferior wall in a single sagittal slice with the greatest EATthick, as previously suggested [17]. This value was expressed in millimiters (mm) and evaluated by the same two nuclear medicine physicians that assessed EATvol.

### Statistical analysis

Continuous variables were expressed as mean ± standard deviation (SD) while categorical variables were expressed as frequencies with specific percentage. Intraclass correlation coefficient (ICC) was used to calculate concordance and to evaluate the reproducibility of the assessment of EATvol and EATthick between the two readers. The average of the values for EATvol and EATthick extracted by the two readers was used for further analyses. T-test and Chi-square test were used to compare different characteristics between two groups of patients based on MFR in the case of normally distributed variables; non-normally distributed variables, assessed with Shapiro-Wilk test, were compared with the Mann–Whitney U test. Spearman’s rank correlation coefficient was used to assess the correlation between MFR and sMBF and other continuous variables. Logistic and linear regression analyses were used when appropriate for the identification of independent variables for MFR and sMBF. This was first performed with a univariate analysis, followed by a multivariable model for factors with a significant association. The aforementioned analyses were performed considering the entire cohort, the group of patients with a medical history of CAD, and subjects without CAD. Receiver Operating Characteristic (ROC) curve was used to evaluate the performances of EATvol and EATthick. A p-value < 0.05 was considered as statistically significant for all the analysis. MedCalc Software version 18.1 for Windows (Ostend, Belgium) was used to perform all statistical analyses.

## Results

### Patients characteristics

**Fig. 1:**
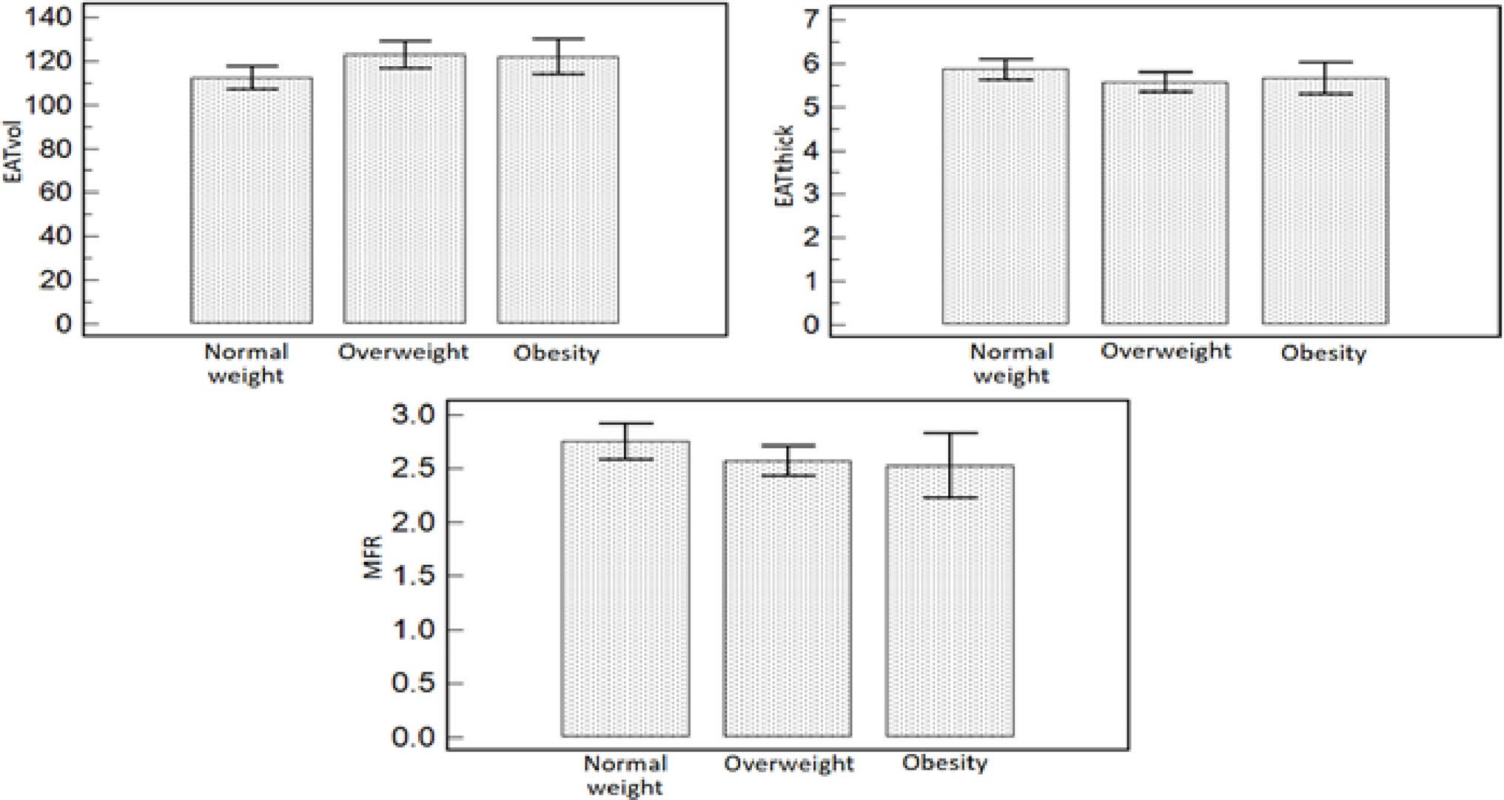
Bar graphs comparing mean values (with 95% confidence interval) of EATvol, EATthick and MFR in the different classes of normal weight patients, overweight subjects and obese patients

A total of 164 patients were included in the study, 106 (64.6%) were men. Mean age was 65 ± 11 while mean body mass index (BMI) was 26.7 ± 4.7. Diabetes was present in 79 (48.2%) of the subjects, 91 (55.5%) patients had hypertension, 77 (47.0%) had hypercholesterolaemia, 78 (47.6%) had a smoking history, 82 (50.0%) had a familiar history of CAD while 36 (22.0%) had a personal history of CAD. In addition, mean EATvol and mean EATthick were 118.5 ± 24.3 and 5.7 ± 1.0, respectively. A summary of the characteristics of the patients of our cohort can be found in Table 1. No statistically significant correlations between BMI and EATvol (p 0.180) or EATthick (p 0.435) were reported. Conversely, MFR decreased significantly from normal patients to overweight subjects and to obese patients (p 0.041, mean MFR values 2.75, 2.57 and 2.53, respectively)(Fig. 1).Furthermore, a good concordance in the assessment of EATvol and EATthick between the two readers was revealed by ICC analysis (ICC 0.867 [IC: 0.820–0.900] and 0.764 [IC: 0.680–0.830], respectively).

**Table 1:** Characteristics of the 164 patients included in the study

Characteristic	Number (%)
Sex	
Male	106 (64.6%)
Female	58 (35.4%)
Age (mean ± SD)	65 ± 11
BMI (mean ± SD)	26.8 ± 4.7
Diabetes	
Yes	79 (48.2%)
No	85 (51.8%)
Hypertension	
Yes	91 (55.5%)
No	73 (44.5%)
Hypercholesterolaemia	
Yes	77 (47.0%)
No	87 (53.0%)
Smoking history	
Yes	78 (47.6%)
No	86 (52.4%)
Familiar history of CAD	
Yes	82 (50.0%)
No	82 (50.0%)
CAD history	
Yes	36 (22.0%)
No	128 (78.0%)
‍Revascularization	
‍ CABG	6 (3.6%)
‍ PTCA	28 (17.1%)
‍MPI findings	
‍ Ischemia	11 (8.1%)
‍ Scar	4 (2.9%)
‍ Ischemia and scar	6 (4.4%)
EATvol (median-IQR)	118 (100–134)
EATthick (median-IQR)	5.7 (5.0–6.5.0.5)

BMI: body mass index; CAD: coronary artery disease; EATvol: epicardial adipose tissue volume; EATthick: epicardial adipose tissue thickness; SD: standard deviation; CABG: coronary artery bypass grafting; PTCA: percutaneous transluminal coronary angioplasty; MPI: myocardial perfusion imaging; IQR: interquartile range

### Relationship between MFR and clinicopathological characteristics

The differences in clinicopathological conditions compared to MFR in different groups are presented in Table 2.

**Table 2:** Differences in clinicopathological characteristics compared to MFR

	MFR < 2	MFR ≥ 2	*p*-value
All the patients			
Age	70 ± 8	64 ± 11	0.007
BMI	27.7 ± 4.4	26.5 ± 4.7	0.198
Diabetes (yes/no)	8/21	71/64	0.010
Hypercholesterolaemia (yes/no)	17/12	60/75	0.160
Hypertension (yes/no)	14/15	77/58	0.380
Smoking history (yes/no)	14/15	64/71	0.930
Familiar history of CAD (yes/no)	14/15	68/67	0.830
CAD history (yes/no)	18/11	110/25	0.219
EATvol	133.7 ± 29.4	115.2 ± 21.9	0.001
EATthick	6.2 ± 1	5.6 ± 0.9	0.004
Patients without CAD history			
Age	71 ± 8	63 ± 12	0.032
BMI	29 ± 4	26 ± 5	0.108
Diabetes (yes/no)	4/14	57/53	0.040
Hypercholesterolaemia (yes/no)	12/6	50/60	0.090
Hypertension (yes/no)	8/10	62/48	0.340
Smoking history (yes/no)	10/8	55/55	0.660
Familiar history of CAD (yes/no)	8/10	54/56	0.710
EATvol	133.7 ± 29.4	115.8 ± 22.2	0.011
EATthick	6.2 ± 1.0	5.6 ± 0.9	0.026
Patients with CAD history			
Age	70 ± 7	64 ± 10	0.091
BMI	26.5 ± 4.0	26.5 ± 3.4	0.968
Diabetes (yes/no)	4/7	14/11	0.270
Hypercholesterolaemia (yes/no)	5/6	10/15	0.750
Hypertension (yes/no)	6/5	15/10	0.750
Smoking history (yes/no)	4/7	9/16	0.980
Familiar history of CAD (yes/no)	6/5	14/11	0.930
EATvol	141.4 ± 28.7	116.4 ± 23.9	0.012
EATthick	6.4 ± 0.6	5.5 ± 1.0	0.007

BMI: body mass index; CAD: coronary artery disease; EATvol: epicardial adipose tissue volume; EATthick: epicardial adipose tissue thickness; SD: standard deviation; MFR: myocardial flow reserve

**Fig. 2:**
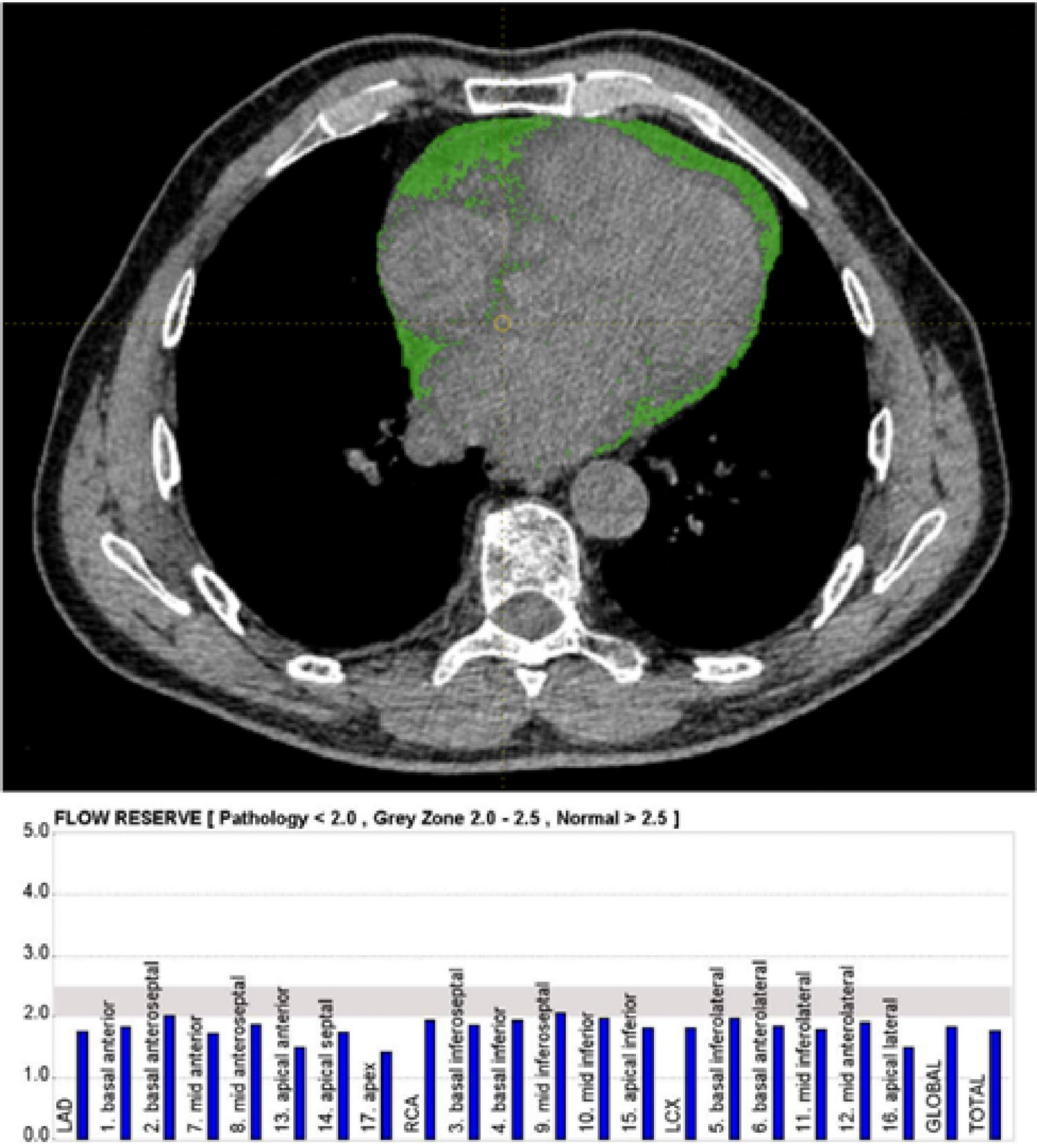
A representative case of a patient with EATvol value of 155 cm^3^, EATthick value of 6.64 mm and an MFR of 1.62

In the total cohort of patients age (p 0.007), presence of diabetes (p 0.010), EATvol (p 0.001) and EATthick (p 0.005) were significantly different between patients with a MFR < 2 and subjects with MFR ≥ 2. The same parameters were significantly different between the two groups even when focusing only on patients without CAD history (p 0.032, 0.040, 0.017 and 0.038 for age, diabetes, EATvol and EATthick, respectively). When considering only subjects with CAD history, only EATvol and EATthick were significantly different between those with impaired MFR and those with preserved values (p 0.010 and 0.007, respectively). A representative case is described in Fig. 2.

### Correlation between different clinicopathological characteristics

**Fig. 3:**
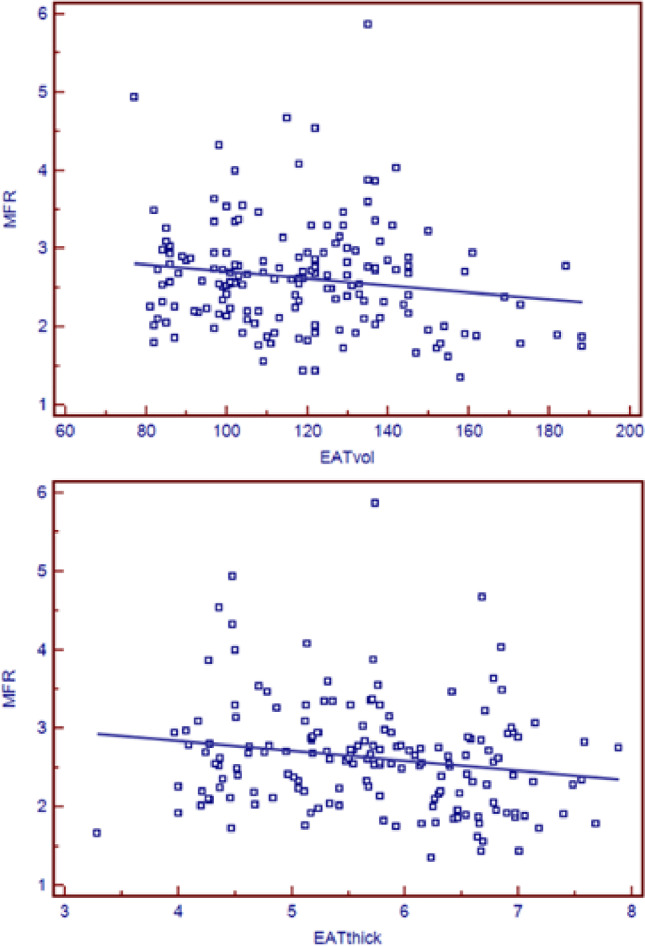
Correlation of MFR with EATvol and EATthick in the total cohort of patients

The analysis of the correlation between different clinicopathological characteristics are presented in Table 3. In the total cohort of patients, significant negative correlations between BMI and MFR (ρ −0.210, p 0.006), BMI and sMBF (ρ −0.338, *p* < 0.001), BMI and rMBF (ρ −0.153, p 0.050) and EATthick with MFR (ρ −0.177, p 0.023) were demonstrated; significant positive correlations were revealed between BMI and EATvol (ρ 0.167, p 0.030), EATvol and EATthick (ρ 0.171, p 0.029) and EATthick with rMBF (ρ 0.171, p 0.029)(Fig. 3). Focusing on the group of patients without a previous history of CAD, significant negative correlation was reported between BMI and MFR (ρ −0.292, p 0.001) and BMI with sMBF (ρ −0.222, p 0.012). Lastly, positive correlations between BMI and EATvol (ρ 0.332, p 0.049) and EATvol with EATthick (ρ 0.419, p 0.013) and a negative correlation between EATthick and MFR (ρ −0.431, p 0.012) were demonstrated for patients with CAD history.

**Table 3:** Correlation between different clinicopathological features

	ρ	*p*-value
All the patients		
BMI - MFR	−0.210	0.006
BMI - EATvol	0.167	0.030
BMI - EATthick	−0.790	0.312
BMI - sMBF	−0.338	< 0.001
BMI - rMBF	−0.153	0.050
EATvol - MFR	−0.132	0.912
EATvol - EATthick	0.171	0.029
EATvol - sMBF	−0.040	0.605
EATvol - rMBF	0.080	0.308
EATthick - MFR	−0.177	0.023
EATthick - sMBF	0.012	0.878
EATthick - rMBF	0.171	0.029
Patients without CAD history		
BMI - MFR	−0.292	0.001
BMI - EATvol	0.130	0.141
BMI - EATthick	−0.127	0.153
BMI - sMBF	−0.222	0.012
BMI - rMBF	−0.057	0.524
EATvol - MFR	−0.081	0.360
EATvol - EATthick	0.098	0.267
EATvol - sMBF	−0.010	0.912
EATvol - rMBF	0.120	0.177
EATthick - MFR	−0.086	0.331
EATthick - sMBF	−0.084	0.344
EATthick - rMBF	0.128	0.149
Patients with CAD history		
BMI - MFR	0.003	0.954
BMI - EATvol	0.332	0.049
BMI - EATthick	0.126	0.456
BMI - sMBF	−0.239	0.157
BMI - rMBF	−0.064	0.705
EATvol - MFR	−0.285	0.091
EATvol - EATthick	0.419	0.013
EATvol - sMBF	0.047	0.789
EATvol - rMBF	0.161	0.341
EATthick - MFR	−0.431	0.012
EATthick - sMBF	0.094	0.578
EATthick - rMBF	0.242	0.152

BMI: body mass index; EATvol: epicardial adipose tissue volume; EATthick: epicardial adipose tissue thickness; MFR: myocardial flow reserve

### Prediction of coronary vascular function

Logistic regression analyses using MFR as dependent variable are presented in Table 4. In the total cohort of patients age (p 0.009), presence of diabetes (p 0.017), presence of CAD history (p 0.025), EATvol (*p* < 0.001) and EATthick (p 0.002) were significant predictors of MFR at univariable analysis. At subsequent multivariable evaluation age (p 0.036), presence of diabetes (p 0.029) and EATvol (0.008) were confirmed as independent predictors. In the group of patients without CAD history age (p 0.037), presence of diabetes (p 0.027), EATvol (p 0.022) and EATthick (p 0.043) were again predictors at univariable analysis and diabetes (p 0.042) and EATvol (p 0.038) were confirmed as affordable predictors after multivariable evaluation. Focusing on patients with CAD history, only EATvol (p 0.021) and EATthick (p 0.016) were significant predictors at univariate analysis, while none of them was confirmed as an independent predictor. ROC curve analyses revealed that the highest AUC values for EATvol and EATthick with MFR were found in the group of patients with CAD history, with high specificity but low sensitivity. These results can be found in Table 5.

**Table 4:** Logistic regression analyses for clinicopathological features and MFR

	Univariable analysis		Multivariable analysis	
	Odds ratio (95% CI)	*p*-value	Odds ratio (95% CI)	*p*-value
All the patients				
Age	−0.0594 (0.0229 − 0.0094)	0.009	0.9290 (0.9117–0.9563)	0.036
BMI	0.9489 (0.8756–1.0284)	0.208		
Diabetes	2.9121 (1.2059–7.0322)	0.017	2.6758 (1.9966–7.1846)	0.029
Hypercholesterolaemia	0.5647 (0.2504–1.2735)	0.168		
Hypertension	1.4444 (0.6365–3.1788)	0.390		
Smoking history	0.9658 (0.4327–2.1556)	0.932		
Familiar history of CAD	0.0838 (0.4095–0.8379)	0.837		
Gender	0.5441 (0.2091–1.3137)	0.168		
CAD history	0.2200 (0.0497–0.9744)	0.025	0.3027 (0.0581–1.2828)	0.052
EATvol	0.9700 (0.9536–0.9868)	< 0.001	0.9741 (0.9564–0.9921)	0.008
EATthick	0.4671 (0.2902–0.7518)	0.002	0.6134 (0.3702–1.1161)	0.066
Patients without CAD history				
Age	0.9529 (0.9116–0.9960)	0.037	0.9608 (0.9159–1.0078)	0.078
BMI	0.9494 (0.8727–1.0328)	0.116		
Diabetes	2.2676 (0.9407–5.4661)	0.027	2.3032 (0.8443–6.2830)	0.042
Hypercholesterolaemia	0.6771 (0.2947–1.5558)	0.102		
Hypertension	1.4359 (0.6246–3.3012)	0.349		
Smoking history	0.8571 (0.3740–1.9642)	0.663		
Familiar history of CAD	1.1384 (0.4973–2.6059)	0.715		
Gender	0.6957 (0.2555–1.8937)	0.414		
EATvol	0.9671 (0.9496–0.9848)	0.022	0.9684 (0.9497–0.9875)	0.038
EATthick	0.5291 (0.3344–0.8372)	0.043	0.7554 (0.4627–1.2333)	0.224
Patients with CAD history				
Age	1.0272 (0.9469–1.1143)	0.099		
BMI	1.0965 (0.8920–1.3477)	0.966		
Diabetes	6.4286 (0.6852–60.3152)	0.283		
Hypercholesterolaemia	0.1556 (0.0166–1.4595)	0.760		
Hypertension	5.1909 (0.6740–24.8296)	0.706		
Smoking history	0.5294 (0.0997–2.8108)	0.983		
Familiar history of CAD	3.6417 (0.5862–21.3975)	0.936		
Gender	4.1905 (0.7491–23.4423)	0.789		
EATvol	1.0148 (1.0076–1.0551)	0.021	1.0154 (0.9751–1.0574)	0.156
EATthick	0.4932 (0.1520–1.2697)	0.016	0.4313 (0.1450–1.2825)	0.093

BMI: body mass index; EATvol: epicardial adipose tissue volume; EATthick: epicardial adipose tissue thickness; MFR: myocardial flow reserve; CAD: coronary artery disease

**Table 5:** ROC curve analysis for EATvol and eatthick with MFR

	Criterion	Sensitivity	Specificity	AUC
All the patients				
EATvol	120.0	47.4	69.0	0.554
EATthick	5.6	48.1	72.4	0.591
Patients without CAD history				
EATvol	108.0	94.5	38.9	0.638
EATthick	5.7	80.0	61.1	0.671
Patients with CAD history				
EATvol	145.0	48.0	100.0	0.742
EATthick	6.4	68.0	90.9	0.767

EATvol: epicardial adipose tissue volume; EATthick: epicardial adipose tissue thickness; MFR: myocardial flow reserve; AUC: area under the curve

The results of linear regression analyses with sMBF as dependent variable are presented in Table 6. When evaluating all the patients BMI, gender and CAD history (*p* < 0.001 for all of them) were significant predictors at univariable analysis and were subsequently confirmed as independently able to predict sMBF (p value 0.005, 0.008 and < 0.001, respectively). BMI (p 0.009) and familiar history of CAD (0.026) were significant predictors at univariable evaluation in the group of patients without CAD history, however only BMI was independently associated with sMBF (p 0.026). Lastly, no clinicopathological parameters resulted as independent predictors in the group of patients with CAD history.

**Table 6:** Linear regression analyses for clinicopathological features and sMBF

	Univariable analysis		Multivariable analysis	
	β (SE)	*p*-value	β (SE)	*p*-value
All the patients				
Age	−0.006 (0.004)	0.132		
BMI	−0.038 (0.009)	< 0.001	−0.021 (0.007)	0.005
Diabetes	0.129 (0.094)	0.170		
Hypercholesterolaemia	0.030 (0.094)	0.750		
Hypertension	−0.108 (0.094)	0.254		
Smoking history	0.138 (0.094)	0.144		
Familiar history of CAD	0.086 (0.094)	0.360		
Gender	−0.440 (0.092)	< 0.001	−0.205 (0.076)	0.008
CAD history	0.926 (0.088)	< 0.001	0.821 (0.087)	< 0.001
EATvol	−0.001 (0.001)	0.3863		
EATthick	0.035 (0.049)	0.4731		
Patients without CAD history				
Age	−0.003 (0.003)	0.237		
BMI	−0.019 (0.007)	0.009	−0.016 (0.007)	0.029
Diabetes	0.108 (0.068)	0.119		
Hypercholesterolaemia	0.030 (0.069)	0.660		
Hypertension	0.029 (0.070)	0.678		
Smoking history	0.054 (0.069)	0.435		
Familiar history of CAD	0.152 (0.068)	0.026	0.117 (0.068)	0.090
Gender	−0.003 (0.003)	0.237		
EATvol	−0.0007 (0.001)	0.572		
EATthick	−0.031 (0.034)	0.365		
Patients with CAD history				
Age	−0.002 (0.002)	0.390		
BMI	−0.007 (0.006)	0.201		
Diabetes	0.024 (0.065)	0.709		
Hypercholesterolaemia	−0.068 (0.062)	0.279		
Hypertension	−0.044 (0.062)	0.482		
Smoking history	0.080 (0.061)	0.200		
Familiar history of CAD	0.064 (0.065)	0.332		
Gender	−0.078 (0.098)	0.433		
EATvol	0.000 (0.001)	0.803		
EATthick	0.011 (0.028)	0.676		

BMI: body mass index; EATvol: epicardial adipose tissue volume; EATthick: epicardial adipose tissue thickness; MFR: myocardial flow reserve; sMBF: stress myocardial blood flow

Evaluation of the influence of EATthick and EATvol on coronary vascular function was also explored by correcting these values for BMI, obtaining therefore EATvol/BMI and EATthick/BMI (Tables 7 and 8). Regarding MFR, these parameters were confirmed as independent predictors only in the total cohort of patients while for sMBF EATthick/BMI was an affordable in the total cohort and in the group of patients without CAD history.

**Table 7:** Logistic regression analyses for clinicopathological features and MFR considering EATvol/BMI and EATthick/BMI

	Univariable analysis		Multivariable analysis	
	Odds ratio (95% CI)	*p*-value	Odds ratio (95% CI)	*p*-value
All the patients				
Age	−0.0594 (0.0229 − 0.0094)	0.009	0.9382 (0.8934–0.9852)	0.010
Diabetes	2.9121 (1.2059–7.0322)	0.017	3.1795 (1.2367–8.1740)	0.016
Hypercholesterolaemia	0.5647 (0.2504–1.2735)	0.168		
Hypertension	1.4444 (0.6365–3.1788)	0.390		
Smoking history	0.9658 (0.4327–2.1556)	0.932		
Familiar history of CAD	0.0838 (0.4095–0.8379)	0.837		
Gender	0.5441 (0.2091–1.3137)	0.168		
CAD history	0.2200 (0.0497–0.9744)	0.025	2.9284 (1.1245–7.6259)	0.027
EATvol/BMI	0.6699 (0.4641–0.9669)	0.031	0.6449 (0.4344–0.9574)	0.029
EATthick/BMI	0.0093 (0.0000–14.2769)	0.211		
Patients without CAD history				
Age	0.9529 (0.9116–0.9960)	0.037	0.9474 (0.8974–1.0002)	0.051
Diabetes	2.2676 (0.9407–5.4661)	0.027	3.5750 (1.0898–11.7276)	0.036
Hypercholesterolaemia	0.6771 (0.2947–1.5558)	0.102		
Hypertension	1.4359 (0.6246–3.3012)	0.349		
Smoking history	0.8571 (0.3740–1.9642)	0.663		
Familiar history of CAD	1.1384 (0.4973–2.6059)	0.715		
Gender	0.6957 (0.2555–1.8937)	0.414		
EATvol/BMI	0.8770 (0.5601–1.3731)	0.568		
EATthick/BMI	0.7515 (0.0001–5.0932)	0.942		
Patients with CAD history				
Age	1.0272 (0.9469–1.1143)	0.099		
Diabetes	6.4286 (0.6852–60.3152)	0.283		
Hypercholesterolaemia	0.1556 (0.0166–1.4595)	0.760		
Hypertension	5.1909 (0.6740–24.8296)	0.706		
Smoking history	0.5294 (0.0997–2.8108)	0.983		
Familiar history of CAD	3.6417 (0.5862–21.3975)	0.936		
Gender	4.1905 (0.7491–23.4423)	0.789		
EATvol/BMI	0.3371 (0.1437–0.7909)	0.004	0.0000 (0.0000–24.4079)	0.225
EATthick/BMI	0.0000 (0.0000–0.0424)	0.012	0.4487 (0.1773–1.1354)	0.090

BMI: body mass index; EATvol: epicardial adipose tissue volume; EATthick: epicardial adipose tissue thickness; MFR: myocardial flow reserve; CAD: coronary artery disease

**Table 8:** Linear regression analyses for clinicopathological features and sMBF considering EATvol/BMI and EATthick/BMI

	Univariable analysis		Multivariable analysis	
	β (SE)	*p*-value	β (SE)	*p*-value
All the patients				
Age	−0.006 (0.004)	0.132		
Diabetes	0.129 (0.094)	0.170		
Hypercholesterolaemia	0.030 (0.094)	0.750		
Hypertension	−0.108 (0.094)	0.254		
Smoking history	0.138 (0.094)	0.144		
Familiar history of CAD	0.086 (0.094)	0.360		
Gender	−0.440 (0.092)	< 0.001	−0.346 (0.093)	0.003
CAD history	0.926 (0.088)	< 0.001	0.270 (0.107)	0.012
EATvol/BMI	0.084 (0.043)	0.053		
EATthick/BMI	2.821 (0.845)	0.001	2.518 (0.791)	0.002
Patients without CAD history				
Age	−0.003 (0.003)	0.237		
Diabetes	0.108 (0.068)	0.119		
Hypercholesterolaemia	0.030 (0.069)	0.660		
Hypertension	0.029 (0.070)	0.678		
Smoking history	0.054 (0.069)	0.435		
Familiar history of CAD	0.152 (0.068)	0.026	0.053 (0.103)	0.605
Gender	−0.003 (0.003)	0.237		
EATvol/BMI	0.135 (0.048)	0.006	0.039 (0.055)	0.474
EATthick/BMI	3.943 (0.909)	< 0.001	3.512 (1.061)	0.001
Patients with CAD history				
Age	−0.002 (0.002)	0.390		
Diabetes	0.024 (0.065)	0.709		
Hypercholesterolaemia	−0.068 (0.062)	0.279		
Hypertension	−0.044 (0.062)	0.482		
Smoking history	0.080 (0.061)	0.200		
Familiar history of CAD	0.064 (0.065)	0.332		
Gender	−0.078 (0.098)	0.433		
EATvol/BMI	−0.045 (0.076)	0.557		
EATthick/BMI	−2.975 (1.689)	0.087		

BMI: body mass index; EATvol: epicardial adipose tissue volume; EATthick: epicardial adipose tissue thickness; MFR: myocardial flow reserve; sMBF: stress myocardial blood flow

## Discussion

Our findings indicate that EATvol is an independent predictor of impaired MFR, in particular in the group of patients without a previous history of CAD. It is known that MPI is particularly useful for the evaluation of subjects with suspected or known CAD, enabling the acquisition of critical prognostic information to guide patient management [11]. On these bases, different predictors have been investigated in the past as early identifiers and reliable markers of impaired MFR. In this setting, our findings are in concordance with similarly published papers performed on populations with various clinical indications. Janik et al. [25] demonstrated that EATvol was an independent predictor of ischemia on [^82^Rb]Rb perfusion PET/CT imaging. Otaki et al. [16] revealed that an increased EATvol indexed to body surface area improved the identification of impaired global MFR and, similarly, Nakazato et al. [26] reported that the same parameter might be related to the presence of both ischemia at [^82^Rb]Rb PET/CT and confirmed stenosis at invasive coronary angiography. More recently, Nappi et al. [19] demonstrated that EATvol predicted reduced MFR evaluated again with [^82^Rb]Rb PET/CT in patients with suspected CAD and normal myocardial perfusion, confirming that visceral pericardium fat may influence coronary vascular function. Interestingly and in contrast with our findings, in their cohort EATvol was also able to predict sMBF. The authors hypothesized that the potential relationship between EATvol and coronary function might have diagnostic implications at initial stage of coronary vascular impairment, facilitating the formulation of appropriate therapeutic strategies. In this regard, our results appear to support this hypothesis, as EATvol was found to predict a reduced MFR in the overall cohort of patients included in the study. However, when stratified by the presence of history of prior CAD, this predictive capacity was confirmed only in subjects without previous CAD. The relatively small sample size of the CAD subgroup (*n* = 36) represents an important methodological limitation that substantially reduces the statistical power of the multivariable analyses performed. As a consequence, the ability to detect meaningful associations or to draw robust conclusions within this subgroup is inherently constrained. This limitation should be explicitly acknowledged when interpreting the results, as estimates derived from such a limited number of cases are more susceptible to instability and may not fully capture the underlying relationships. Therefore, findings pertaining to the CAD subgroup must be considered exploratory in nature and interpreted with particular caution.

Compared with the aforementioned studies, our findings explore novel aspects. In particular, [^13^N]NH3 offers higher extraction efficiency and more accurate MBF quantification, particularly at high flow rates, allowing for a more precise assessment of microvascular function and regional perfusion heterogeneity and permitting calculation of absolute MBF, MFR, and myocardial flow patterns under both rest and stress conditions, which may provide incremental diagnostic and prognostic insight over [^82^Rb]Rb PET imaging [27].

In our study, EATthick was not able to predict impaired sMBF or MFR. Different findings were however reported by Alam et al. [18] who underlined that this parameter, but not EATvol, was an independent predictor of impaired MFR in patients with non-obstructive coronary artery disease assessed with [^82^Rb]Rb PET/CT. In addition, the Authors reported an excellent inter-observer agreement for the evaluation of EATthick, an insight confirmed in our paper and proved also when assessing EATvol. In our study, while both EATvol and EATthick were significantly different between groups with impaired and preserved MFR in univariate analysis, only EATvol emerged as a robust independent predictor of impaired MFR in the multivariate model for the overall cohort and the no-CAD subgroup. This suggests that the volumetric assessment of EAT may provide a more comprehensive and independent measure of its pathophysiological impact on coronary microvascular function compared to a single linear measurement of thickness. Furthermore, the ROC curve analysis revealed that the predictive performance (AUC) of both EATvol and EATthick for impaired MFR was modest in the overall cohort. The highest AUC values were observed in the small subgroup of patients with CAD history, exhibiting high specificity but low sensitivity. This indicates that while elevated EAT measures are a specific marker of microvascular dysfunction in advanced patients, they have limited value as a standalone screening tool and are best interpreted within a multifactorial risk model.

The deposition of EAT can be measured with different modalities such as echocardiography, which shows good reproducibility but is affected by the lack of the quantification of total load, and CT or magnetic resonance which allow an accurate volumetric quantification with higher reproducibility [28]– [29]. Different published studies allowed recent literature to support the emerging idea that the evaluation of EAT not only allows the identification of impaired MFR, but could also be useful as a prognosticator with potential therapeutic implications [5]– [6, 29–31]. In particular, the concept of a potential interplay between inflammatory stimuli released from EAT and MFR before the clinical manifestations of CAD has emerged. The presence of dysfunctional EAT may be linked to early plaque development, inflammation, adverse cardiac events and worst outcome [28, 32–36]. It was also observed that pericardial adipose tissue accumulation is closely associated with CAD morbidity, more than any other body fat distribution [35]. In this setting, the anatomical contiguity between EAT, myocardium and coronary arteries supports the idea of a paracrine interaction between these structures, making the first a more likely predictor of left ventricular mass compared to general measures of adiposity [37]. Interestingly, correlations between EATvol and different clinicopathological factors have been reported in the past such as, for example, degree of myocardial fibrosis, left ventricle systolic function and BMI [19, 38]. We reported correlations of EATvol with BMI and EATthick with MFR, even though after being found in the total cohort, they were only confirmed in the group of patients with CAD history.

Growing evidences supporting the fact that EAT can be considered as a metabolically active organ and an important source of pro- and anti-inflammatory mediators and cytokines are emerging. These are believed to apply local effect of surrounding coronaries and myocardium [36]. In addition, these markers have been implicated in the clinical pathogenesis of ventricular remodelling, underlying therefore the possible importance of EAT as a marker of cardiovascular risk. EAT may impair coronary microvascular function through both paracrine and mechanical mechanisms, by secreting pro-inflammatory cytokines, adipokines, and reactive oxygen species that can induce endothelial dysfunction, vascular inflammation, and impaired vasodilation in adjacent coronary microvessels. Additionally, increased EAT volume may exert local compressive effects on the coronary microcirculation, further limiting perfusion reserve and contributing to microvascular dysfunction. Additionally, EAT has been associated with increased fibrosis and structural remodeling of the microvascular bed, which can further compromise coronary flow reserve [39]. Collectively, these biochemical and mechanical interactions contribute to microvascular dysfunction, potentially leading to myocardial ischemia even in the absence of obstructive epicardial coronary artery disease. Interestingly, it has been recently proposed that EAT may influence cardiac and vascular function by the production of adipokines through endocrine-paracrine signaling [40]. However, a deeper understanding of the clear mechanism of action and signaling and the interactions between EAT and the myocardium needs to be reached, since a protective pathway starting from the heart to EAT has also been proposed [41]. Even though in concordance with previously published literature, our results currently lack a clear and immediate clinical relevance, but support a possible role of EAT quantification in the risk stratification of patients. In this setting, future research needs to focus on larger and multicentric samples to answer to these needs and to find a defined threshold of EATvol which may have a clinical impact for a better stratification of patients’ risk. As already underlined, the implementation of risk stratification by including the assessment of EAT is able to significantly improve the reclassification of subjects with impaired MFR [16].

The clinical implications of our findings, even if in concordance with other papers, remains exploratory. EAT quantification could be integrated into clinical decision-making and multimodality imaging workflows as an additional biomarker for cardiovascular risk and microvascular dysfunction. EAT can be non-invasively measured using CT, MRI, or PET/CT, providing information on both volume and tissue characteristics such as density or metabolic activity. Incorporating EAT assessment alongside functional imaging modalities, such as PET myocardial perfusion, stress MRI, or coronary flow reserve evaluation, could help identify patients at higher risk of coronary microvascular dysfunction, even in the absence of obstructive coronary artery disease. This integrated approach may inform personalized management strategies, including closer monitoring, targeted lifestyle or pharmacologic interventions, and risk stratification for preventive therapies.

Several limitations affect our study and need to be acknowledged. First, this is a retrospective, monocentric investigation. Second, and importantly, the cohort of patients with a history of CAD was relatively small (36/164, 22%), which limits the statistical power of the subgroup analyses. This likely explains why, despite strong univariate associations, EATvol and EATthick were not confirmed as independent predictors of MFR in this specific group. The findings in this subgroup should therefore be interpreted with caution and require validation in larger, dedicated studies. Moreover, the absence of prognostic information regarding EATvol and EATthick values and MPI results are another important limitation.

## Conclusion

In conclusion, we reported some correlations for EATvol and impaired MFR evaluated with [^13^N]NH3. Our findings confirm the idea that EAT may influence coronary function and may be a potential early predictor of coronary dysfunction, as previously suggested on the basis of perfusion [^82^Rb]Rb PET imaging.

## Data Availability

the datasets generated during and/or analysed during the current study are available from the corresponding author on reasonable request.
